# COMMON FEATURES OF AREA AGENCIES ON AGING AND IMPLICATIONS FOR AGING SERVICE INNOVATIONS

**DOI:** 10.1093/geroni/igae098.0645

**Published:** 2024-12-31

**Authors:** Traci Wilson

**Affiliations:** USAging, Washington, District of Columbia, United States

## Abstract

Established by the Older Americans Act (OAA) in 1973, Area Agencies on Aging (AAAs) across the country assess local community needs and plan, coordinate, and deliver a range of long-term services and supports that help older adults and people with disabilities to age well at home. There is a commonly heard phrase, “if you’ve seen one AAA, you’ve seen one AAA”, but is this helpful or true? Based on an analysis of organizational-level data from USAging’s 2022 National Survey of AAAs (74 percent response rate), this paper delineates typologies of AAAs based on characteristics such as organizational structure, funding sources, agency size, location and how this relates to the types of services offered by AAAs, populations served, and their involvement in initiatives such as age-friendly communities or housing and homelessness prevention. The findings illustrate that all AAAs deliver a core set of OAA-funded services and supports, and funding sources outside of the OAA, including state and local funding, Medicaid Waiver, and contracts with health care entities provide opportunities to deliver additional services to more older adults. While state regulations largely determine the organizational structure and setting of AAAs, there is evidence that each typology of AAA innovates, collaborates and delivers high-quality aging services and supports. By understanding these commonalities, researchers, practitioners, advocates and policy makers can better support the local organizations that support community-living older adults across the country.

